# Childhood sensorineural hearing loss and adult mental health up to 43 years later: results from the HUNT study

**DOI:** 10.1186/s12889-019-6449-2

**Published:** 2019-02-08

**Authors:** Mariann Idstad, Kristian Tambs, Lisa Aarhus, Bo Lars Engdahl

**Affiliations:** 1 Department of Chronic Diseases and Ageing, Division for Mental and Physical Health, Norwegian Institute of Public Health, Pb 4404 Nydalen, 0403 Oslo, Norway; 2 Department of Occupational Medicine and Epidemiology, The National Institute of Occupational Health, Norway, Pb 8149 Dep, 0033 Oslo, Norway

**Keywords:** Sensorineural hearing loss, Mental health, Depression, Anxiety, Subjective well-being, Self-esteem, Longitudinal, Children, Childhood, Adults

## Abstract

**Background:**

Hearing loss is a global public health problem putting millions of people at risk of experiencing impediments in communication and potentially impaired mental health. Many studies in this field are based on small, cross sectional samples using self-report measures. The present study aims to investigate the association between childhood sensorineural hearing loss and mental health in adult men and women longitudinally in a large cohort with a matched control group, and hearing is measured by pure-tone audiometry. Studies of this kind are virtually non-existing.

**Methods:**

The present study combines data from two large studies; the School Hearing Investigation in Nord-Trøndelag (SHINT) carried out yearly from 1954 to 1986, and the second wave of the Nord-Trøndelag Health Study (HUNT 2) conducted from 1995 to 1997. The participants were 7, 10 or 13 years during the SHINT, and between 20 and 56 years old during HUNT 2. The total sample consisted of 32,456 participants (of which 32,104 in the reference group). Participants with a sensorineural hearing loss in SHINT of 41 dB or more were classified with moderate-severe hearing loss (*N* = 66), 26–40 dB as mild (N = 66) and 16–25 dB as slight (*N* = 220). Mental health in adulthood was measured in HUNT 2 by symptoms of anxiety and depression, subjective well-being, and self-esteem. The association between childhood sensorineural hearing loss and adult mental health was tested by means of ANOVA.

**Results:**

There was a significant relation between slight childhood sensorineural hearing loss and lowered subjective well-being in women (B = −.25, *p* = 0.038). Further investigation of the results revealed a significant association between slight hearing loss and symptoms of anxiety and depression (B = .30, *p* = 0.054) and between mild hearing loss and lowered self-esteem (B = .63, *p* = 0.024) among women aged 20–39 years. There were no significant relations between childhood sensorineural hearing loss and any of the three mental health outcomes among men.

**Conclusions:**

This study suggests that women with slight or mild sensorineural hearing loss from childhood experience elevated levels of symptoms of anxiety and depression, lowered subjective well-being and lowered self-esteem. However, the results should be interpreted with caution due to a lack of power in some analyses.

## Background

Hearing loss is a global public health problem, listed by the World Health Organization as one of the leading causes of disability worldwide [1]. An estimated 32 million children and 328 million adults suffer from hearing loss, corresponding to 5.3% of the world’s population [2]. Using data from 29 countries, Stevens and colleagues [3] reported that 1.4% of children and 9.8–12.2% of adults suffered from hearing loss, and that prevalences are especially high in low- and middle-income countries. Sensorineural hearing loss (SNHL) is a result of damage to the auditory nerve or the hair cells of the inner ear, and may be acquired, genetic or idiopathic. About 1–4 per 1000 babies are born with SNHL [4–6]. Although it is generally assumed that a more severe hearing loss entails a greater developmental setback for the child, this is a complex matter depending on many factors like for example the child’s age at identification [7].

One of the most important repercussions of any hearing loss is the impediment of communication [8]. The inability to communicate with other people may lead to feelings of isolation and frustration, and, ultimately, to poor mental health. Like hearing loss, poor mental health is a major public health issue. Depression and anxiety disorders rank among the top six contributors to global disability and tend to be more common among women than among men [9]. Subjective well-being (the extent to which an individual evaluates his or her life as good and desirable) and self-esteem are also important markers of mental health since they are associated with symptoms of anxiety and depression [10, 11]. Hence, if hearing loss negatively impacts a person’s self-esteem, this may be detrimental to his or her relationships with other people as well as quality of life [12].

A number of studies have demonstrated an association between hearing loss and mental health in children and adolescents. Recently, Theunissen and colleagues [13] reviewed the literature on psychopathology in children and adolescents with hearing loss. Studies in which the participants had permanent bilateral hearing loss of 40–120 dB in the better ear were included in the review. The authors concluded that compared to peers with normal hearing, children and adolescents with hearing loss are at a higher risk of developing mental health problems. Similarly, another recent review including a wide variety of definitions of hearing loss reported that children with hearing loss had scores on emotional and behavioural difficulties that were one quarter to one third of a standard deviation higher than their hearing peers [14]. There is also evidence for a longitudinal association. For example, Hogan and colleagues [15] found that children with hearing loss at baseline had elevated levels of adverse psychosocial outcomes like emotional distress and peer problems 6 years later. In this study, however, parents were simply asked a categorical yes or no question about whether their child had hearing problems. The type or severity of hearing loss could thus not be identified. Moeller [12] reviewed studies on psychosocial development in children focusing especially on children with mild to moderately severe sensorineural hearing losses. The tendency was for children with hearing loss to score lower on quality of life and higher on behaviour problems than hearing children, but results varied and the majority of the studies did not detect any association between the degree of hearing loss and psychosocial outcomes. However, the author noted that this might be due to low statistical power since these studies were based on small samples.

Although there seems to be a growing body of research demonstrating a relationship between hearing loss and mental health and self-esteem, results are not unanimous. Mejstad and colleagues [16] invited all children between 11 and 18 years who had received a hearing aid in three counties in Southern Sweden to partake in a questionnaire survey. A total of 111 children participated (43%), of which 60 pupils attended regular schools and had mild to moderate hearing loss, 23 attended schools for the hard of hearing and had moderate to severe hearing loss, and 28 attended schools for the deaf and had profound hearing loss. Although the exact degree of hearing loss was unknown, the type of school served as a proxy, since children with more severe hearing losses are normally referred to special schools like the two latter ones. Questionnaire data on mental health and self-esteem showed that boys had more mental health problems than girls and that children with profound hearing loss had lower scores on mental health and self-esteem than the other two groups. Since the study lacked a control group, the authors compared the mental health scores and the self-esteem scores with results from two Nordic population based studies. They detected a lower score on mental health among the Swedish girls, but no difference between the boys. As a group, there was no difference in self-esteem between Swedish children and those in the Nordic studies, however, the children in regular Swedish schools had higher scores and children in deaf Swedish schools had lower scores.

Finally, Øhre and colleagues [17] examined existing research on the prevalence of mental disorders among prelingually deaf adults and concluded that although symptoms of anxiety and depression seem to be more common among the deaf, studies are simply too few, too heterogenous and of too poor quality to draw any firm conclusions.

To sum up, there is broad agreement among researchers that hearing loss is a public health problem that may have detrimental effects, that it is a complex and understudied issue, and that more research is needed [17–20]. Despite the magnitude of this problem, population based studies are rare [3], and many studies rely on self-report measures of hearing loss.

The aim of the present study is to investigate the relation between childhood sensorineural hearing loss and mental health in adult men and women, respectively, in terms of subjective well-being, self-esteem, and symptoms of anxiety and depression. In a previous paper based on cross-sectional data, we found a weak to moderate association between hearing loss and mental health in adults [21], and now we use that same data material together with data on schoolchildren to investigate longitudinal effects of childhood sensorineural hearing loss. This is an epidemiological study spanning 43 years. To the best of our knowledge, it is the first study of its kind.

## Methods

### Sample

The present study combines baseline data from the School Hearing Investigation in Nord-Trøndelag (SHINT) with follow-up data from the Nord-Trøndelag Hearing Loss Study (NTHLS) and the second wave of the Nord-Trøndelag Health Study (HUNT 2) in Norway. All NTHLS participants also participated in the HUNT 2. Altogether, data from three questionnaires are used in this study: the questionnaire from the NTHLS (NTHLS-Q1), and both questionnaires from HUNT 2 (HUNT2-Q1 and HUNT2-Q2). The HUNT2-Q2 was handed out at the examination cite and returned by mail later, the response rate for the sample used in the present study was 76.3%.

The SHINT was an audiometric screening of all schoolchildren attending regular schools in the county of Nord-Trøndelag aged 7, 10 and 13 years from 1954 to 1986. The late H.F. Fabritius who was a Norwegian Ear, Nose and Throat (ENT) specialist, led the investigation. Great efforts were made to ensure participation, and Dr. Fabritius himself even rowed a boat to reach a small island off the Norwegian coast in order to include the people who lived there. Unfortunately, the exact number of participants is unknown, since records were only made for children with hearing loss, and not for children with normal hearing. We do know, however, that 78,524 children were born in Nord-Trøndelag between 1941 and 1977, which may serve as a proxy.

The initial screening included as good as every single pupil in the entire county and took place in a quiet location at the respective school. A trained hearing assistant or a nurse performed the hearing examination. Air-conduction thresholds were obtained by means of pure tone audiometry at 0.25, 0.5, 1, 2, 4 and 8 kHz utilizing Amplivox audiometers (type 70, and later models 51 and 81). Pupils were registered with hearing loss if 1) thresholds of 20 dB or greater at three or more frequencies in the same ear were detected, or if 2) a threshold of 30 dB or more at one or more frequencies were detected.

A total of 10,269 children were classified with hearing loss at the screening. All of these children were then invited to a full examination by an ENT specialist at one of the 14 out-patient clinics in Nord-Trøndelag. Questionnaire data regarding the children’s ear problems were collected from the parents. The ENT specialist performed a new pure tone audiometry with both air- and bone-conduction thresholds as well as a complete medical examination including family and medical history, recording findings and diagnoses. Children underwent one or more ENT examinations depending on the diagnoses in order to ensure correct classification. Dr. Fabritius defined SNHL as hearing loss in which the air-conduction thresholds followed those of the bone-conduction, although he did not include a maximum accepted air-bone gap in this definition. In this study, we rely on the diagnosis made by Dr. Fabritius and the other ENT specialists. The attendance rate was 97% between 1954 and 1962 and it is likely that the high participation rate persisted [22].

Out of the 10,269 children who tested positively for hearing loss at the screening, 1489 were diagnosed with Sensorineural Hearing Loss according to Dr. Fabritius’ definition. However, only 3066 out of the 10,269 children from the screening in the SHINT also participated in the NTHLS as adults, and just 462 out of the original 1489 SNHL cases from the SHINT also participated in the NTHLS. There were several reasons for this attrition, like for example loss of identification number or not being old enough to be invited to the NTHLS, or possibly moving away from the county (for more details, see [23]).

For the purpose of the present study, we wanted to distinguish between profound-severe, moderate, and mild hearing loss, respectively. We estimated the average hearing threshold of 0.5, 1, 2 and 4 kHz in both ears from the last audiogram (from the ENT examination, not from the screening), which for most participants was at age 13. This means that the hearing loss might have emerged at different points in time for the participants, somewhere between birth and 13 years of age. We defined moderate-severe hearing loss as 41 dB or more (ranging to 100, which means that this group also includes profound hearing loss), mild hearing loss as 26–40 dB, and slight hearing loss as 16–25 dB, resulting in 67 cases in each of the two former groups and 223 cases in the latter. This reduced the case group from 462 to 357.

Finally, since the present study has mental health variables as outcome, we wanted to exclude cases that might be struggling with mental health problems at baseline. We cross-checked the case group with data on the following conditions registered at baseline: “Retarded”, “Cerebral paralysis”, “Mental health issues”, “Mental distress/ depressed”, “Down’s syndrome”, and “Is receiving psychological treatment”. Three individuals were registered as “Retarded”, one individual was registered with “Cerebral paralysis”, and one individual with “Downs syndrome”. These cases were excluded from further analysis, resulting in a total case group of 357 of which 220 are in the case group with slight hearing loss whereas the other two groups include 66 individuals each.

The NTHLS was a part of the second wave of the Nord-Trøndelag Health Study (HUNT 2) carried out in 1995–97. In HUNT 2, the entire adult population in Nord-Trøndelag County was invited to participate, whereas in NTHLS, the adult population in 17 of the 23 municipalities was invited to participate. Data on mental health were available for 51,574 people (62.8%) and the age span was 20 to 101 years. Participants in the NTHLS who had not been diagnosed with a hearing loss at SHINT were used as the reference group, following the assumption that all of these people grew up in Nord-Trøndelag and therefore participated in the SHINT. Since the SHINT lasted from 1954 to 1986 and the NTHLS from 1995 to 1997, the oldest participants to attend both SHINT and NTHLS would have been 13 years in 1954, thus 56 years old in 1997. Therefore, we only selected people 56 years and younger from the NTHLS for the reference group. This resulted in a sample of 32,456 individuals.

To sum up, the sample consisted of 66 individuals with moderate-severe hearing loss, 66 with mild hearing loss, 220 with slight hearing loss, and 32,104 with normal hearing (reference group).

### Measures

#### Childhood sensorineural hearing loss (predictor)

For the purpose of the present study, only children diagnosed with SNHL were selected. In the present study, we estimated the average hearing threshold of 0.5, 1, 2 and 4 kHz in both ears from the last audiogram. We defined moderate-severe hearing loss as 41 dB or more, mild hearing loss as 26–40 dB, and slight hearing loss as 16–25 dB.

#### Mental health (outcome)

Ten of the 25 items from the Symptom Checklist-25 [24], here called SCL-10, were included in the NTHLS-Q1 and were used to measure mental health. Four questions tap anxiety and six questions tap depression. The distribution of the SCL-10 scores was skewed and the scores were therefore log transformed. A high score on this index reflects poor mental health. Cronbach’s alpha was .86. Using another available data material [25], we estimated the correlation between the SCL-25 global score (anxiety and depression jointly) and the short-form global score to .97.

#### Subjective well-being (outcome)

The index consists of three items from HUNT2-Q1, phrased as follows: When you think about your life at the moment, would you say that you are by and large satisfied with life, or are you mostly dissatisfied? (seven response categories ranging from “very happy” to “very unhappy”); In the course of the last 2 weeks, have you been feeling safe and calm?; In the course of the last 2 weeks, have you been feeling happy and optimistic? (four response categories ranging from “no” to “a lot” for both items). The first question was recoded so that a high score on this variable reflects a high level of subjective well-being. Because of the different number of response categories, the items were standardized before they were added into a sum score indicator. Cronbach’s alpha was .84.

#### Self-esteem (outcome)

Four items from The Rosenberg Self-Esteem Scale [26] were included in HUNT2-Q2. The questions are phrased as follows: I take a positive attitude toward myself; I feel that I am a person of worth, at least on an equal plane with others; I feel I do not have much to be proud of; I certainly feel useless at times. The two last items were recoded before the items were added as a sum score so that a high score on this variable reflects a low level of self-esteem. Cronbach’s alpha was estimated to.74 in the present data set. The four-item short form scale has been shown to correlate .95 with the original instrument [21].

The scores for all of the three outcome variables were standardized before entered in the analyses.

#### Control variables

Control variables included in this study are age, education, mother’s education and father’s education, respectively. The data were provided by Statistics Norway with the following ten categories: 1) No education/preschool, 2) primary school 1st to 7th grade, 3) middle school 8th to 10th grade, 4) high school 11th to 12th grade, 5) high school diploma 13th grade, 6) high school extended, 7) college or university, lower level, 14th to 17th grade, 8) college or university, higher level, 18th to 19th grade, 9) PhD level, 20th grade or more, 10) education not reported. We recoded categories 1, 2, 3 and 10 into “Primary school”, category 4 into “middle school”, categories 5 and 6 into “high school”, category 7 into “college/university, less than 4 years”, and categories 8 and 9 into “college/university, 4 years or more”.

### Treatment of missing values

As mentioned earlier, records for normal hearing were not registered in the SHINT, which means that for many frequencies, values below 20 dB were missing. The missing value for each frequency was therefore replaced by the frequency specific mean values of the scores below 20 dB in the original sample (*N* = 10,269).

For the outcome variables, we used SPSS Missing Value Analysis (MVA), expectation maximization (EM) for imputation of missing data where the respondent had valid data on at least half of the items. The ten SCL items were used to predict each other. This reduced missing values from 7.9 to 0.3%. For SWB, the items were used to predict each other in those cases where the respondent had valid data on two of the three questions, reducing missing values from 8.0 to 4.9%. The Self-esteem items were included in HUNT2-Q2, and, as mentioned above, this questionnaire was returned by somewhat fewer respondents (76.3%) than the other two questionnaires. For this reason, there was a larger percentage of missing values on the Self-esteem variable compared to the SCL-10 and SWB variables. Missing data were replaced for respondents who had valid data on at least two of the four items. The four Self-esteem items were used to predict each other, reducing missing values from 19.6 to 17.8%.

A total of 319 participants (0.9%) as well as 4697 fathers (14.5%) and 3402 mothers (14.5%) did not report level of education and missing values were replaced by the sample mean.

### Design and statistical analyses

This study applies a longitudinal design, investigating the association between childhood sensorineural hearing loss at baseline and adult mental health up to 43 years later. In order to study this association separately in men and women, we split the dataset into to new datasets; one including men only, and one with women only. Three ANOVA analyses (IBM SPSS 24, General Linear Models, Unianova) were conducted consecutively in each data set (the total sample, the male sample and the female sample, respectively) with Childhood Sensorineural Hearing Loss (CSNHL) as the predictor and SCL-10, Subjective Well-Being, and Self-Esteem as the respective outcomes. Since the dependent variables were standardized before entered in the analyses, the unstandardized regression coefficients (b) show adjusted group mean differences scaled in fractions of a standard deviation. This makes it easier to interpret the results. The first model tests the association between childhood sensorineural hearing loss and adult mental health, whereas the second model tests the same association controlled for age, education, and mother’s and father’s education, respectively. In the analyses with the total sample we also included sex as a control variable (but not in the analyses with the male sample or female sample).

## Results

Descriptive statistics for the three case groups with childhood sensorineural hearing loss (slight, mild, and moderate-severe) and for the reference group without childhood sensorineural hearing loss, respectively, are presented in Table 1. The table shows that the average scores on symptoms of anxiety and depression, subjective well-being and self-esteem are quite similar in the case groups and the reference group.

**Table 1:** Characteristics of the four groups: the reference group with no childhood sensorineural hearing loss (CSNHL) and the case groups with slight, mild, or moderate-severe CSNHL

	No CSNHL		Slight CSNHL		Mild CSNHL		Moderate-severe CSNHL	
	Men (*N* = 15,059)	Women (*N* = 17,045)	Men (*N* = 147)	Women (*N* = 73)	Men (*N* = 33)	Women (*N* = 33)	Men (*N* = 40)	Women (*N* = 26)
Age: mean (SD)	40.0 (10.1)	39.5 (10.1)	36.9 (8.5)	36.2 (9.0)	38.9 (9.3)	35.5 (10.0)	36.3 (9.1)	37.4 (8.6)
Education: N (%)								
Primary school	2978 (19.8)	3919 (23.0)	34 (23.1)	19 (26.0)	5 (15.1)	7 (21.2)	6 (15.0)	5 (19.2)
Middle school	6655 (44.2)	5945 (34.87)	72 (49.0)	25 (34.2)	15 (45.6)	14 (42.4)	21 (52.5)	12 (46.2)
High school	1815 (12.0)	2813 (16.0)	17 (11.6)	13 (17.8)	5 (15.1)	10 (30.3)	4 (10.0)	6 (23.1)
College/university < 4 years	2142 (14.2)	2821 (16.6)	15 (10.2)	12 (16.5)	5 (15.1)	0	6 (15.0)	2 (7.7)
College/university > 4 years	1469 (9.8)	1547 (9.1)	9 (6.1)	4 (5.5)	3 (9.1)	2 (6.1)	3 (7.5)	1 (3.8)
Father’s education: N (%)								
Primary school	13,390 (89.0)	15,039 (88.2)	129 (87.8)	68 (93.2)	28 (84.9)	30 (91.0)	33 (82.5)	24 (92.4)
Middle school	790 (5.2)	930 (5.5)	12 (8.2)	3 (4.1)	1 (3.0)	1 (3.0)	6 (15.0)	1 (3.8)
High school	645 (4.3)	782 (4.6)	3 (2.0)	2 (2.7)	4 (12.1)	1 (3.0)	1 (2.5)	0
College/university < 4 years	215 (1.4)	268 (1.6)	3 (2.0)	0	0	1 (3.0)	0	0
College/university > 4 years	19 (0.1)	26 (0.2)	0	0	0	0	0	1 (3.8)
Mother’s education: N (%)								
Primary school	14,177 (94.2)	16,035 (94.1)	143 (97.3)	71 (97.3)	32 (97.0)	32 (97.0)	39 (97.5)	25 (96.2)
Middle school	204 (1.4)	244 (1.4)	1 (0.7)	0	0	0	0	0
High school	637 (4.2)	719 (4.2)	3 (2.0)	2 (2.7)	1 (3.0)	1 (3.0)	1 (2.5)	1 (3.8)
College/university < 4 years	21 (0.1)	26 (0.2)	0	0	0	0	0	0
College/university > 4 years	20 (0.1)	21 (0.1)	0	0	0	0	0	0
SCL-10, range 10–40: mean (SD)	12.08 (3.2)	12.95 (3.8)	12.17 (3.4)	13.88 (4.4)	12.25 (2.6)	13.71 (5.4)	12.62 (3.4)	13.00 (3.1)
SWB, range 3–15: mean (SD)	11.50 (2.1)	11.56 (2.2)	11.47 (2.1)	11.05 (2.4)	11.82 (1.9)	11.47 (2.3)	12.00 (1.9)	11.79 (2.1)
SE, range 4–16: mean (SD)	7.03 (1.9)	7.66 (1.9)	7.02 (2.0)	7.97 (1.8)	7.29 (2.5)	8.13 (2.4)	6.73 (1.6)	7.52 (1.7)

The unadjusted and adjusted results from the ANOVA analyses are presented in Tables 2, 3 and 4. Table 2 shows the results from the analysis of the total sample. No significant effects were detected.

**Table 2:** ANOVA: Relation between childhood sensorineural hearing loss (CSNHL) and symptoms of anxiety and depression (SCL-10), subjective well-being (SWB), and self-esteem (SE) in adulthood for the total sample. *N* = 32,456 (of which 32,104 are in the reference group)

	Slight CSNHL (N = 220)			Mild CSNHL (N = 66)			Moderate-severe CSNHL (N = 66)		
Outcome ^a^	b (CI) ^b^	p	N cases	b (CI) ^b^	p	N cases	b (CI) ^b^	p	N cases
SCL-10 unadjusted	.04 (−.09–.18)	.536	214	.09 (−.16–.34)	.478	63	.04 (−.21–.30)	.740	63
SCL-10 adjusted	.11 (−.03–.24)	.128	214	.11 (−.14–.36)	.391	63	.10 (−.16–.35)	.456	63
SWB unadjusted	−08 (−.22–.05)	.235	207	.03 (−.22–.28)	.832	61	.19 (−.06–.43)	.131	65
SWB adjusted	−.11 (−.24–.03)	.116	207	.00 (−.24–.25)	.972	61	.16 (−.08–.40)	.201	65
SE unadjusted	−.05 (−.19–.10)	.544	178	.21 (−.08–.50)	.148	46	−.16 (−.44–.11)	.240	52
SE adjusted	−.03 (−.11–.18)	.653	178	.21 (−.07–.49)	.148	46	−.11 (−.37–.16)	.420	52

^a^Adjusted scores = adjusted for sex, age, education, father’s education, and mother’s education

^b^Unstandardized regression coefficient (b) with 95% confidence interval (CI). The coefficients show mean deviations from individuals without hearing loss in fractions of a standard deviation

**Table 3:** ANOVA: Relation between childhood sensorineural hearing loss (CSNHL) and symptoms of anxiety and depression (SCL-10), subjective well-being (SWB), and self-esteem (SE) in adulthood for men. *N* = 15,279 (of which 15,059 are in the reference group)

	Slight CSNHL (*N* = 147)			Mild CSNHL (*N* = 33)			Moderate-severe CSNHL (*N* = 40)		
Outcome ^a^	b (CI) ^b^	p	N cases	b (CI) ^b^	p	N cases	b (CI) ^b^	p	N cases
SCL-10 unadjusted	.05 (−.11–.20)	.577	143	.10 (−.23–.43)	.550	32	.11 (−.19–.41)	.481	39
SCL-10 adjusted	.05 (−.11–.21)	.515	143	.11 (−.23–.44)	.524	32	.12 (−.18–.42)	.428	39
SWB unadjusted	−01 (−.18–.15)	.866	140	.15 (−.22–.52)	.419	28	.25 (−.06–.55)	.116	40
SWB adjusted	−.04 (−.20–.12)	.645	140	.13 (−.23–.50)	.464	28	.20 (−.10–.50)	.197	40
SE unadjusted	−.03 (−.21–.15)	.759	118	.12 (−.30–.54)	.568	21	−.16 (−.51–.19)	.373	30
SE adjusted	−.01 (−.19–.16)	.904	118	.11 (−.30–.52)	.577	21	−.12 (−.46–.23)	.512	30

^a^Adjusted scores = adjusted for age, education, father’s education, and mother’s education

^b^Unstandardized regression coefficient (b) with 95% confidence interval (CI). The coefficients show mean deviations from individuals without hearing loss in fractions of a standard deviation

**Table 4:** ANOVA: Relation between childhood sensorineural hearing loss (CSNHL) and symptoms of anxiety and depression (SCL-10), subjective well-being (SWB), and self-esteem (SE) in adulthood for women. *N* = 17,177 (of which 17,045 are in the reference group)

	Slight CSNHL (*N* = 73)			Mild CSNHL (N = 33)			Moderate-severe CSNHL (*N* = 26)		
Outcome ^a^	b (CI) ^b^	p	N cases	b (CI) ^b^	p	N cases	b (CI) ^b^	p	N cases
SCL-10 unadjusted	.21 (−.03–.46)	.089	71	.10 (−.27–.48)	.584	31	.05 (−.37–.48)	.814	24
SCL-10 adjusted	.22 (−.03–.46)	.083	71	.10 (−.27–.47)	.587	31	.05 (−.37–.47)	.816	24
SWB unadjusted	−22 (−.46–.03)	.080	67	−.08 (−.42–.27)	.659	33	.10 (−.30–.50)	.620	25
SWB adjusted	−.25 (−.49 – -.01)	.038	67	−.10 (−.45–.24)	.543	33	.09 (−.31–.48)	.683	25
SE unadjusted	.13 (−.13–.38)	.328	60	.30 (−.09–.69)	.138	25	−.07 (−.49–.35)	.743	22
SE adjusted	.13 (−.12–.38)	.285	60	.27 (−.11–.65)	.161	25	−.09 (−.49–.32)	.696	22

^a^Adjusted scores = adjusted for age, education, father’s education, and mother’s education

^b^Unstandardized regression coefficient (b) with 95% confidence interval (CI). The coefficients show mean deviations from individuals without hearing loss in fractions of a standard deviation

Table 3 shows the results from the analysis of the male sample. There were no significant associations between childhood sensorineural hearing loss and any of the three outcomes.

Table 4 shows the results from the analysis of the female sample. The table shows that there was a significant association between slight childhood sensorineural hearing loss and lowered subjective well-being in women. There was also a clear trend for lowered self-esteem among women with mild hearing loss as well as a higher symptom level of anxiety and depression among women with slight hearing loss, but these results did not reach significance, although effect sizes were similar to that of subjective well-being (B = .27, *p* = 0.161, and B = .22, *p* = 0.083, respectively).

We took the analysis one step further by dividing the sample of women into two different age cohorts; one aged 20–39 years and one aged 40–56 years. Rerunning the analyses in each age cohort revealed a significant association between slight hearing loss and symptoms of anxiety and depression (B = .30, *p* = 0.054) as well as a significant association between mild hearing loss and lowered self-esteem (B = .63, *p* = 0.024) among women in the youngest age group. Although the associations between slight hearing loss and self-esteem among women in the youngest age group and between slight hearing loss and lowered subjective well-being among women in the oldest age group had small to moderate effect sizes, they did not reach significance (B = .26, *p* = 0.134 and B = −.35, *p* = 0.083, respectively).

## Discussion

The aim of the present study was to investigate the relation between sensorineural hearing loss in childhood and mental health in adulthood in terms of symptoms of anxiety and depression, subjective well-being and self-esteem. The results in this study suggest that women in general who have a slight childhood sensorineural hearing loss experience lower subjective well-being as adults compared to hearing women. Furthermore, younger adult women with slight hearing loss seem to be vulnerable to an elevated symptom level of anxiety and depression and younger adult women with mild hearing loss report lower self-esteem than their hearing peers. These results offer support to the growing body of literature demonstrating that people with childhood hearing loss are at risk of experiencing poor mental health (i.e., [13–15]). However, we did not find any effects in the male cohort, which may be interpreted as evidence that males with hearing loss are not at any particular risk of mental health problems in adulthood. This result is also in line with the finding from the study by Mejstad and colleagues [16] that the Swedish boys did not differ from their Nordic peers. Furthermore, the Swedish girls had poorer mental health than their Nordic peers, and this resembles our result of (young adult) women with slight or mild hearing loss scoring significantly poorer on all three mental health outcomes. However, in the Swedish study, the children with the most severe hearing loss had the lowest scores on mental health and self-esteem, whereas in our study, it was the opposite: those classified with moderate-severe hearing loss did not differ from the reference group; the only significant effects were found in the groups with slight or mild hearing loss. This is in contrast to the prevailing notion that greater severity of the hearing loss is accompanied by correspondingly greater difficulties, and supports the idea that it is equally important to focus on people with less severe hearing loss [7]. The fact that we did not detect any effects in the group classified with moderate-severe hearing loss in our study is, however, somewhat counterintuitive. One might expect deaf individuals to feel completely excluded from the hearing world, whereas people with slight or mild hearing loss might be assumed to feel like they could be a part of it to a greater extent. But it may also be the opposite; maybe it is more exhausting to have limited hearing and to always try and hear what people say than to be in the stable condition of hearing close to nothing. Finally, if deaf people who consider themselves as part of a deaf minority culture are less likely to report low subjective well-being, this might have deflated our results. These are however mere speculations, and the results should be interpreted with caution due to the small number of participants in some of the analyses.

It is difficult to explain why we did not detect any effects among the men with hearing loss in our study. It is possible that this reflects a gender difference in which women with hearing loss struggle more when they enter adulthood. Perhaps women find the impediments that the hearing loss puts on communication more burdensome than men, or perhaps this merely reflects the tendency for women in general to score higher on anxiety and depression than men [9].

### Strengths and limitations

The size of the SHINT (all the schools in Nord-Trøndelag County were included) and NTHLS/HUNT (more than 50,000 respondents) investigations and the possibility to follow the children with hearing loss all the way into adulthood more than 40 years later makes our data material rather unique. No other study has been able to do this, as far as we know. Since children were screened for hearing loss at three different ages (7, 10 and 13 years), most cases have probably been identified. Another strength is the use of pure tone audiometry, which leaves little room for measurement error. Obviously, hearing measurement equipment did not hold the same standard in the 1950’s as in the 1980’s, however, the researchers used the best equipment following international standards at the time. What is more, the children were thoroughly examined one or more times by an ENT specialist using air- and bone conduction thresholds. Hence, we feel reasonably sure that most cases were indeed identified. However, although all efforts were made to ensure correct identification of sensorineural hearing loss, the occurrence of misclassification cannot be completely ruled out. Also, we do not know if the children received hearing aids of any kind.

The recording of additional disorders and problems in the SHINT allowed us to exclude individuals who had been registered with mental health problems at baseline from our study. This means that we can be reasonably sure that the children included in our study were relatively mentally healthy at baseline. We know that the researchers who collected the data in the SHINT were meticulous in their work. Furthermore, it is not very likely that the children in our study struggled with many severe challenges other than the hearing loss, since children with multiple challenges are normally referred to special schools.

Although it is an advantage to be able to follow up a cohort over more than forty years, it is also a challenge. Firstly, many things may have happened in between baseline and follow-up; people may get a mental illness and still have plenty of time to recover without us knowing. Even if our failing to detect a relation between hearing loss and mental health among men reflects a true absence of associations, our results do not necessarily mean that boys with hearing loss automatically learned to cope with their hearing loss and then uninhibitedly reached adulthood with their mental health intact. We cannot know for sure. Secondly, it might have been very different growing up with sensorineural hearing loss in the 1950’s than in the 1980’s. There is a larger acceptance and understanding for minority groups of all kinds now that hopefully makes it somewhat easier for people with hearing loss to navigate in the hearing world. But following such a line of reasoning, one might have expected more mental health problems among the eldest group of adults and not the youngest, but that was not the case. Rather, if it is the youngest adults that struggle the most, then this is especially important to address in our (seemingly) accepting society. On the other hand, maybe the eldest group struggles just as much, only we did not have the statistical power to detect it.

There was a loss of observations from the SHINT to the NTHLS, as only 3066 of the 10,269 original cases participated in the NTHLS. Our research group investigated this in a previous article [23] and found several explanations. Many SHINT participants were not invited to the NTHLS because they lived in other municipalities, some were too young to be invited, and some were lost because of missing identification numbers. Moreover, no important differences were found between SHINT participants who did and did not attend the NTHLS. For more details, see Aarhus et al. [23]. In general, there is a tendency for prelingually deaf people not to participate in epidemiological studies because researchers may not possess the necessary procedures to reach signing people, moreover, limited literacy might also be an issue [17]. As in all epidemiological studies, there is always a danger of systematic bias and attrition.

Our reference group consisted of adults who participated in the NTHLS and who were not registered with hearing loss at the SHINT. This means that we assume that these people went to primary school in Nord-Trøndelag and, consequently, that they attended SHINT. This is a rather crude approximation. The reference group undoubtedly includes some false negatives since some of the respondents in this group may have moved to Nord-Trøndelag as young adults, and therefore did not attend the SHINT. However, this would not have affected our results much since even a substantial number of false negatives in the reference group would have been greatly outnumbered by the true negatives.

## Conclusion

Our study suggests that girls with slight or mild SNHL are at risk of having mental health problems as adults. On the one hand, this supports earlier research showing an association between hearing loss and mental health in general, but on the other hand, it contradicts it, since we only detected effects for women and not for men. Studies on hearing loss are vulnerable to attrition and our study is no exception; our results therefore need to be interpreted with caution. This field of research is still in great need of more longitudinal studies based on large, representative samples in order to disentangle the many complex issues related to hearing loss and mental health.

## Abbreviations

HUNT 2: The second wave of the Nord-Trøndelag Health Study, Norway
NTHLS: the Nord-Trøndelag Hearing Loss Study, Norway
SHINT: the School Hearing Investigation in Nord-Trøndelag, Norway
SNHL: Sensorineural Hearing Loss
